# Saliva: What Dental Practitioners Should Know about the Role of This Biofluid in the Transmission and Diagnostic of SARS-CoV-2

**DOI:** 10.3390/medicina57040349

**Published:** 2021-04-06

**Authors:** Miguel Angel Casillas Santana, Farid Alonso Dipp Velázquez, Carolina Sámano Valencia, Alan Martínez Zumarán, Norma Verónica Zavala Alonso, Ricardo Martínez Rider, Marco Felipe Salas Orozco

**Affiliations:** 1 Maestría en Estomatología con Opción Terminal en Ortodoncia, Facultad de Estomatología, Benemérita Universidad Autónoma de Puebla, Puebla, Pue. 72410, Mexico; farid.dipp@correo.buap.mx (F.A.D.V.); carolina.samano@correo.buap.mx (C.S.V.); 2 Especialidad en Ortodoncia, Facultad de Estomatología, Univesidad Autónoma de San Luis Potosí, San Luis Potosí, S.L.P. 78290, Mexico; alanzuma@uaslp.mx (A.M.Z.); nveroza@fest.uaslp.mx (N.V.Z.A.); rmrider@uaslp.mx (R.M.R.)

**Keywords:** COVID-19, dental practice, diagnostic methods, saliva, SARS-CoV-2

## Abstract

A novel severe acute respiratory syndrome coronavirus 2 (SARS-CoV-2) outbreak has become a global ongoing pandemic. This pandemic represents a great work risk for all health professionals, it includes dental professionals who are in constant contact with saliva, which represents one of the main routes of transmission of the disease. This is due to the fact that a wide variety of oral tissues and cells are susceptible to infection by SARS-CoV-2 and that they express the ACE2 receptor, which is the main route of entry of the virus into cells, as well as the proteins TMPRSS and furin that contributes to the binding of the virus to the host cells. According to recent studies, some of the oral cells most susceptible to infection by SARS-CoV-2 are the epithelial cells of the salivary glands. This explains the presence of the virus in the saliva of infected patients and provides scientific evidence that supports the use of saliva as a biofluid that offers the opportunity to develop new detection and diagnostic techniques. This is because saliva is much easier to collect compared to nasopharyngeal swab. However, the presence of the virus in saliva, also represents a great source of transmission, since the main form of infection is through microscopic drops that are generated when infected people cough or sneeze. Likewise, health professionals, such as dentists are exposed to contagion through saliva. The objective of this review article is to provide a perspective on the main cells and tissues that can be affected by the virus, the risk of contagion that the presence of the virus in saliva represents for dentists; and the new techniques developed from saliva samples for the diagnosis and surveillance of SARS-CoV-2 infection. This review is expected to contribute to the knowledge of oral health professionals about the risk of saliva in the spread of SARS-CoV-2, but also its advantages as a diagnostic tool for pandemic control. In conclusion, the authors can mention that information that provides more scientific evidence of the mechanisms of infection of the coronavirus in oral cells and tissues is being published continually. This also explains the presence of the virus in the saliva of infected people and the risk of contagion that this means. It also provides scientific evidence of the use of saliva as a biofluid for the detection, diagnosis, monitoring, and control of the spread of the virus.

## 1. Introduction

The coronavirus family was discovered in the 1960s. From this family, so far three types of coronaviruses have caused pandemics worldwide. These three viruses are the severe acute respiratory syndrome coronavirus (SARS-CoV) [1], Middle East respiratory syndrome coronavirus (MERS-CoV) [2], and the second severe acute respiratory syndrome coronavirus (SARS-CoV-2) [3]. These three viruses belong to the genus of beta coronaviruses that can infect both humans and mammals. The SARS-CoV-2 shares 79.5% identity with severe acute respiratory syndrome coronavirus (SARS-CoV) and shares 96% identity with a bat coronavirus (BatCoVRaTG13) [4,5]. The structure of SARS-CoV-2 is similar to SARS-CoV, it has a diameter of ~50–200 nm, sense single-stranded RNA virus and it is made up of four structural proteins, three of them (spike (S), envelope (E), membrane (M)) are responsible for forming the viral envelope. The remaining structural protein, the nucleocapsid (N), carries the RNA genome (~30 kb). The protein S of the virus is a protein responsible for adhesion, fusion, entry, and transmission into host cells by binding with human angiotensin-converting enzyme 2 (ACE2) receptors; nevertheless, the exact mechanism of how these processes occur is still unknown [6]. Since the beginning of the pandemic, the different forms of transmission of the virus have been investigated, and even probabilistic models have been developed and therefore help to improve measures to stop the spread of the virus [5,7].

Although there are constant publications about the effects of the pandemic in dental practice, many studies still need to be conducted, mainly in terms of methods for preventing infections during clinical practice. In addition, the SARS-CoV-2 pandemic represents an unprecedented global public health crisis. Oral health professionals are facing for the first time to know, selecting and applying a challenging and enormous amount of scientific information that emerges as the pandemic advances, to continue with dental care, promote and protect the oral health of the population, while protecting patients, assistants, and dental office workers from the risks posed by the SARS-CoV-2 infection.

For several decades, saliva has been continuously studied as a means of assessing human health because it contains a wide variety of biomarkers. This is because saliva is a mixture of fluids from the salivary glands, crevicular fluids, desquamated epithelial cells, microorganisms, and a great number of proteins such as immunoglobulins, hormones, enzymes, and cytokines [8]. The very diverse composition of saliva allows it to be used in the detection of pathogens, even, in some cases, it exceeds the capacity to detect physical changes compared to serum [9]. Due to the above, saliva is a biofluid with an enormous potential for evaluating the health of individuals. Therefore, it may be a better option than the nasopharyngeal and oropharyngeal swabs used for the detection and diagnosis of SARS-CoV-2 [10,11].

The objective of this review is to summarize the available scientific evidence that explains why saliva is a great risk of contagion for dentists and a better option to detect, monitor, and control the transmission of SARS-CoV-2.

## 2. SARS-CoV-2 Diagnosis

Thanks to the efforts of various groups of researchers, rapid identification and sequencing of the viral genome were possible. It has allowed the fast development of diagnostic tests that enabled better control of the spread of the pandemic as well as its surveillance [12]. The gold standard for the diagnosis of SARS-CoV-2 is the molecular method known as reverse transcription polymerase chain reaction (RT-qPCR) [3], samples for this method are the nasopharyngeal and oropharyngeal swabs or sputum [13]. Another diagnostic method that is applied is the taking of chest radiographs through which the presence of pneumonia can be identified with the following characteristics: multilobar ground-glass opacities with a peripheral, asymmetric, and posterior distribution [14].

## 3. SARS-CoV-2 Pathophysiology

Several studies have been reported around the world based on the experience of China, Italy, Spain, Germany, France, U.S., and other countries. At the beginning of the outbreak, the mean incubation period was 5.2 days and the combined case-fatality rate in China was 2.3%. The principal comorbidities are the presence of coronary artery disease, diabetes, hypertension, and lung disease. Therefore, these illnesses increase the severity and clinical symptoms of SARS-COV-2 infection [15]. As the infection spreads throughout the world, the heterogeneity of the disease makes its study and clinical management more complex, ranging from “asymptomatic patients” with SARS-CoV-2 nucleic acid test positive without any clinical symptoms and signs, to “critical patients” with acute respiratory distress syndrome (ARDS) [16]. Nonetheless, the common clinical features of SARS-CoV-2 include fever, fatigue, dry cough, myalgia, and dyspnea; atypical symptoms, such as headache, dizziness, abdominal pain, diarrhea, nausea, and vomiting, are also observed [17].

The virus propagates and migrates down the respiratory tract along the conducting airways, the life cycle of the virus with the host consists of the following five steps: attachment, penetration, biosynthesis, maturation, and release [18]. ACE2 is the main receptor for both SARS-CoV-2 and SARS-CoV, therefore, overexpression of human ACE2 enhanced the disease severity, and this suggests that viral entry into cells is a critical step in this infectious disease. SARS-CoV-2 does not use other coronavirus receptors such as aminopeptidase N and dipeptidyl peptidase 4. Thus, the SARS-CoV-2 spike protein has a strong binding affinity with the host cell surface ACE2 receptor facilitating the virus entry and replication [19]. Interestingly, Rokni et al. mentioned that after an airborne entry, SARS-CoV-2 leads to infection of ACE2 expressing target cells resulting in high viral replication [20]. High levels of proinflammatory cytokines including IL-2, IL-7, IL-10, G-CSF, IP-10, MCP-1, MIP-1A, and TNFα play a main role in the evolution of the disease. This overexpression of cytosines has been called a “cytokine storm,” and it is considered a major factor in the pathogenesis [21]. The “cytokine storm” may cause immunopathologic injury in the lung that leads to severe pneumonia and the patients are also at risk of developing secondary infections [22]. On the other hand, for SARS-CoV-2, the response to viral infection by type I IFN is suppressed, the production of IFN-α and IFN-β is important for antiviral innate immune response and can suppress viral replication and dissemination at an early stage [20].

## 4. Organs of the Human Body Sensitive to SARS-CoV-2 Infection

Studies focused on determining which cells in the human body can be infected by SARS-CoV-2 have been conducted. Based on the biology of viral infection and clinical disease management, these studies have demonstrated that differences in SARS-CoV-2 severity are associated with the high affinity of SARS-CoV-2 S protein for ACE2, suggesting that populations with higher expression of ACE2 are more susceptible to infection [23,24]. For example, Li et al. compared ACE2 expression levels in 31 human tissues. Likewise, they compared the levels of ACE2 expression between young and older people, and between men and women. They also investigated the correlation between ACE2 expression levels and the degree of immune response [25]. Regarding the levels of expression of ACE2 in human tissues; six human tissues were those that showed the highest levels of expression, these tissues are the heart, adipose tissue, kidneys, testis, thyroid, and small intestine. The tissues that showed medium levels of ACE2 expression were adrenal gland, liver, colon, bladder, and the lungs. Other studies report that cells that most express ACE2 in lung tissue are type II alveolar cells. Finally, some of the tissues that showed low ACE2 expression were skin, muscle, pituitary, and stomach [25].

Although patients infected with SARS-CoV-2 present radiographic images with lung involvement, the lungs only showed a medium level of ACE2 expression [26]. It could be because, despite the low levels of ACE2 expression in the lungs, the respiratory system is the most common route of entry into the body of SARS-CoV-2. Other tissues that showed an expression of SARS-CoV-2 were the testis. It could be one of the factors that may explain the higher degree of infection in men compared to women. The authors also found a correlation between older men and a positive immune response. The positive immune response in this type of patient could give rise to the inflammatory cytokine storm that results in lung injury in patients infected with SARS-CoV-2 [27].

## 5. Oral Cells and Tissues Sensitive to SARS-CoV-2 Infection

Xu et al. investigated oral tissues whose cells could be the target of SARS-CoV-2 infection. They used data extracted from two public databases that contain information about RNA sequences. Likewise, they used their own transcriptome database to determine ACE2 expression levels in cells of the oral cavity. The authors of this study demonstrated that the expression of ACE2 in oral tissues was, from the highest to the lowest: the oral tongue, floor of the mouth, the base of tongue, and other sites. In the study, it was also determined that the cell type of oral tissue with the highest ACE2 expression are epithelial cells [28]. Chen et al. reported that ACE2 expression could be detected in epithelial cells of salivary glands [29]. Song et al. additionally reported that the salivary glands are a sensitive site for SARS-CoV-2 infection [30]. Due to the ability of SARS-CoV-2 to infect the surrounding cells of the salivary glands as well as the glands themselves, it is speculated that this infection can cause various pathologies related to salivary glands, such as sialadenitis, hyposecretion, and sialolithiasis [31]. The detection of SARS-CoV-2 in tissues and oral fluids coincides with what was previously reported for SARS-CoV-1 [32]. Sakaguchi et al. reported that the anatomical areas of the mouth that are sensitive to SARS-CoV-2 infection are: tongue, gingiva, taste buds and saliva. This is due to the fact that these tissues express molecules (ACE2, transmembrane protease [TMPRSS] and furin) that facilitate the entry of SARS-CoV-2 into cells. In the case of saliva, it contains desquamated cells that express the aforementioned molecules [33]. Fernandes Matuck et al. reported the presence of SARS-CoV-2 in periodontal tissues (junctional epithelium, adjacent oral gingival epithelium, and connective tissue) in a post-mortem study [34]. Galicia et al. reported that dental pulp can express ACE2 and TMPRSS2. This will facilitate the infection of the dental pulp by SARS-CoV-2 [35]. Finally, Sawa et al. additionally reported that the tongue expresses a high percentage of molecules that facilitate infection by SARS-CoV-2. In addition to the tongue, the authors mentioned that the lip and cheeks are sites that express entry molecules for SARS-CoV-2 [36].

## 6. Saliva as a Risk of Contagion in Dental Practice

The virus has been detected in various body fluids and secretions such as blood [37], stool [38], gastrointestinal tissue [39], semen [40], tears [41], and saliva [42]. The expression of ACE2 is relatively high in various oral tissues, making the cells that make up these tissues the target of SARS-CoV-2 infection, therefore the manipulation of these tissues would mean a risk of contagion. This risk of contagion is especially significant for members of the dental health area. This high risk is because in dental practice there are some procedures that can produce aerosols. It has been previously reported that dental office aerosols can contain a wide variety of microorganisms such as fungi, bacteria, and viruses [43]. These findings indicate that the oral mucosa and saliva, are a potentially high-risk route of SARS-CoV-2 infection and transmission in dental practice.

Human-to-human airborne transmission is the main transmission route of the SARS-CoV-2. The SARS-CoV-2 air transmission can be divided into two mechanisms, which are saliva drops or aerosols. Saliva droplets are expelled when coughing or sneezing and have a size between 10 and 100 μm. Being relatively large, saliva droplets quickly fall to the ground due to gravity. Due to this, the transmission by this type of mechanism requires a certain physical proximity. The aerosol can also be generated when a person with SARS-CoV-2 coughs, sneezes, and talks, or as in the case of the dental area, they can be generated during the care of patients in which irrigation is included during the use of the high-speed handpiece. Microscopic drops of which the aerosol is composed have a size smaller than 10 µm. Due to their relatively smaller size, microscopic drops can remain suspended in the air or travel long distances through ventilation systems, so transmission through this mechanism does not require that an infected individual be close to a healthy person. A healthy person can become infected with SARS-CoV-2 by ingesting or inhaling small drops of contaminated saliva or if his mucosa have direct contact with contaminated surfaces due to the two aforementioned airborne transmission mechanisms [44]. It has been previously reported that the aerosol generated during dental procedures contains a great diversity of microorganisms (including viruses) and that these can be inhaled by patients or common people, or deposited on the various contact surfaces present in the dental office [43,45]. Van Doremalen et al. reported that SARS-CoV-2 is capable of surviving in aerosols for a period of 3 h, in addition, the half-life of SARS-CoV-2 was 1.2 h [46]. However, the time reported by these authors was in an experimental setting, so it is unknown if the virus’s survival time would be the same or longer in a clinical setting.

Various protocols have been developed for the care of patients who require dental treatment during the pandemic. These protocols have been published and reviewed in previous articles [47,48,49]. Within them, one of the main tools for detecting patients suspected of being infected with SARS-CoV-2 is the application of a questionnaire. It includes questions about whether patients have had contact with people infected with SARS-CoV-2, their travel history, and the most common symptoms present in people infected with SARS-CoV-2. However, although the most common symptoms of SARS-CoV-2 can be easy to detect, asymptomatic infections are possible, and the transmission can occur before the disease symptoms appear, therefore, asymptomatic patients, with mild or uncommon symptoms (such as eye irritation) [50] that occur in young patients or children, may be difficult to identify [51]. To date, a great variety of children infected with SARS-CoV-2 have been reported, it is important to note that the vast majority of them did not need any type of medical treatment [52,53,54].

Kim et al. reported the prevalence of asymptomatic patients with a previous diagnosis of SAR-CoV-2 by RT-PCR. The absence or presence of symptoms was reported through the application of a questionnaire. From the 213 patients interviewed, only 41 patients remained asymptomatic [55]. Kim et al. evaluated the absence or presence of symptoms in 71 patients previously confirmed as SARS-CoV-2 positive. Of these 71 patients, 3 (4.2%) patients were presymptomatic at the time of the study and 10 (14%) patients remained completely asymptomatic. The age range of asymptomatic patients ranged from 8 years to 79 years of age. Five of the ten asymptomatic patients presented comorbidities [56]. The prevalence of asymptomatic patients ranges from 1.2 to 14% [57].

Being aware of reports of uncommon symptoms in patients with SARS-CoV-2 is important for dentists as it will help them recognize suspicious patients before the dental consultation. Furthermore, although it is more common for young patients to remain asymptomatic, the absence of symptoms can also occur in elderly patients [58].

Some reports indicate that patients with SARS-CoV-2-infected pneumonia are healthcare workers (HCWs) [59]. Thus, it is important for dentists to improve prevention protocols and strategies to reduce the generation of aerosols [10].

Recently, some researchers have proposed the use of devices designed to reduce or control the generation of aerosols during dental procedures. However, the use of this type of equipment has not become widespread [60,61]. Additionally, more research is required on the use of high-volume suction and air filtration systems, especially in clinical settings where surgical procedures can generate aerosols (including those from saliva).

## 7. Skin and Oral Manifestations of SARS-CoV-2

The cutaneous manifestations of SARS-CoV-2 have been poorly described. Recently, a group of French researchers described the cutaneous lesions in a group of 14 SARS-CoV-2-positive patients. The different skin lesions described included exanthema, cold urticarial, chickenpox-like vesicles, livedo, violaceous macules, non-necrotic purpura, necrotic purpura, chilblain appearance lesions, eruptive cherry angioma, and chilblain [62]. A team of Spanish researchers also described the skin lesions of 375 Spanish patients. The cutaneous lesions in these patients were vesicles or pustules, other vesicular eruptions, urticarial lesions, maculopapular eruptions, and livedo or necrosis. Vesicular eruptions were the first symptom in 15% of patients [63]. Other cutaneous manifestations reported are urticarial (in some patients it was the first symptom) [64], acral lesions, petechiae [65], rash [66], erythema multiforme-like eruption [67], and varicella-like exanthema [68]. These skin lesions can occur in the back, chest, feet, and hands and can occur in adults, youth, and children [69].

Few reports on the possible oral manifestations of SARS-CoV-2 were published. Chen et al. applied 108 questionnaires to patients previously diagnosed with SARS-CoV-2. Of these 108 questionnaires, 90% (96) were online and the remaining 10% (12) were offline. Of the 108 patients surveyed, 52 of them were men and 56 were women. Although the authors mention that the questionnaire included questions about 14 possible oral symptoms related to SARS-CoV-2 infection, in this article only the results of the most common oral symptoms are reported, these symptoms were amblygeustia, dry mouth, inflammation of mouth, and enlargement of lymph nodes in the submandibular region [29].

Martín Carreras-Presas et al. presented three case reports of patients with oral symptoms related to SARS-CoV-2. The case reports presented in the aforementioned article have certain peculiarities. Of the three patients, only one had a confirmed diagnosis of SARS-CoV-2 infection, of the other two patients, one remained in quarantine at home on the instructions of his doctor since he did not present serious symptoms; and the last patient was suspected of SARS-CoV-2 infection since his wife was confirmed as infected. The intraoral inspection was performed through photographs and only one patient received an intraoral clinical examination. From the three patients, two were men in their 50 s and a woman in their 60 s. The first patient was a 56-year-old male who had moderate symptoms of SARS-CoV-2. The main oral symptoms reported by the patient were lesions similar to recurrent herpetic lesions. The patient had no previous history of herpetic infection. Valaciclovir was administered to the patient to treat the lesions, and these disappeared in a period of 10 days. The second patient was a 58-year-old man. This patient’s wife had been diagnosed with SARS-CoV-2, so the patient and his wife were quarantined at home. The oral symptoms of this patient were pain on the palate accompanied by small ulcers [70]. The patient was prescribed with an antiseptic mouthwash that helped the symptoms disappear in 1 week. The third patient was a 65-year-old woman. She was the only patient who underwent a test for detecting SARS-CoV-2, which was positive. The patient also had hypertension and obesity as risk factors for SARS-CoV-2. This patient had oral and cutaneous symptoms related to SARS-CoV-2. Oral symptoms included desquamative gingivitis, blisters on the lip mucosa, and pain on the tongue. Alternatively, the cutaneous symptoms included eruptions in different parts of the body (back, skin, and genital area) [70].

These three patients developed exanthematous lesions, such as those produced by other types of viral infections (for example, herpes simplex lesions). It is important to consider an intraoral examination of patients diagnosed with SARS-CoV-2 to identify oral lesions related to viral infection that could help the identification of asymptomatic or mildly symptomatic patients. Likewise, it is important to conduct studies in this type of patient to confirm that the lesions are the result of viral infection and not because of stress [70]. All the oral symptoms related to SARS-CoV-2 mentioned here can serve to expand the questionnaire focused on the detection of patients suspected of SARS-CoV-2 before dental care [71,72].

In the recent literature, hypotheses have been proposed to explain the appearance of oral symptoms in SARS-CoV-2 positive patients. Finsterer and Stollberger have proposed that SARS-CoV-2 can infect the peripheral nerves, affecting sensory functions and causing the appearance of symptoms such as ageusia and dysgeusia. In addition, SARS-CoV-2 would travel through axons of peripheral nerves, crossing the blood-brain barrier and infecting the central nervous system (CNS). CNS infection would cause local meningitis that could contribute to the appearance of the aforementioned symptoms [73]. A second theory proposed by the same authors to explain the appearance of ageusia/dysgeusia in patients ineffective by SARS-CoV-2 mentions that the virus would infect the epithelial cells present in the taste buds, altering their function [73]. Another theory proposed by them mentions that SARS-CoV-2 positive patients generate antibodies that can attack the taste buds, altering their function [73]. In their final theory on the appearance of ageusia/dysgeusia in SARS-CoV-2 positive patients, the authors mention that the appearance of the symptoms may be due to side effects caused by the drugs used to combat the virus infection [73].

Vaira et al. propose that ageusia is due to the fact that the infection of the salivary glands by SARS-CoV-2 causes a change in the composition of saliva which causes the taste molecules present in food to degrade rapidly and do not reach the papillae taste buds. The authors also mention that the infection of the olfactory bulb by SARS-COV-2 can also affect the sense of taste due to the close relationship of these two systems [74]. Nataf proposes that ageusia is because the SARS-CoV-2 infection affects the synthesis of neurotransmitters such as histamine, dopamine, and serotonin [75]. On the other hand, Mariz et al. propose that the SARS-CoV-2 infection alters the expression of ACE2, which prevents angiotensin II from being degraded and causing the appearance of dysgeusia [76]. Galvan Casas et al. propose that the appearance of mouth ulcers in SARS-CoV-2 positive patients is due to a co-infection of bacteria, fungi, and other respiratory viruses that cause cell lysis [63]. Sarode et al. propose that the appearance of oral ulcers is thus, SARS-COV-2 infects red blood cells, decreasing the oxygenation of the tissues. Likewise, they also mention that the virus mimics the action of hepcidin causing an increase in ferritin and causing anemia, which would contribute to the appearance of oral ulcers [77]. This theory could also be complemented by the changes in oral microcirculation that SARS-CoV-2 can cause [78].

## 8. Detection of SARS-CoV-2 in Saliva

Due to the recent pandemics caused by the appearance of respiratory viruses, the possibility that respiratory infections caused by viruses can be detected from saliva samples has been investigated. In 2017, To et al. investigated the possibility of detecting respiratory viruses through molecular methods from saliva samples of 258 hospitalized patients suspected of respiratory viral infection. Each patient got nasopharyngeal and saliva samples taken (this sample was taken without any type of special device). Subsequently, the two types of samples were analyzed using molecular methods (immunofluorescence assay and multiplex PCR). The concordance of results between the saliva and nasopharyngeal samples was 83.8%. Therefore, it indicates that saliva sample analysis can help improve clinical management of patients [79].

It has been reported that *Rhesus Macaques* ACE2 works much as human ACE2, making it one of the main routes of infection for coronavirus [80]. Likewise, these types of nonhuman primates, when infected with coronaviruses, showed the same type of lung lesion seen on chest radiographs in humans [81]. In addition, Liu et al. demonstrated that ACE2 is present in high amounts in epithelial cells found in the vicinity of the ducts of salivary glands in an infection model in *Macaca mulatta* [82].

To et al. demonstrated the utility of the use of saliva samples for detecting SARS-CoV-2 through molecular methods (RT-qPCR). In their study, they included 12 patients who had previously been positive for coronavirus and 33 patients who had been negative for coronavirus from nasopharyngeal and sputum samples. The saliva samples of the 45 patients were processed to extract the total of the nucleic acids present in them. After the extraction of nucleic acids, the detection of SARS-CoV-2 was performed through RT-qPCR. All the coronavirus negative patients were confirmed negative from saliva samples. Regarding positive patients, 11 of the 12 saliva tests (91.7%) were also confirmed as positive. In addition, in molecular saliva tests, the gene used to identify the presence of SARS-CoV-2 was the coronavirus S gene [83].

Chen et al. conducted a study confirming the positive diagnosis of SARS-CoV-2 using saliva samples from thirty-one patients. They used the molecular method of RT-PCR for detecting the coronavirus from saliva samples. Of the samples from the thirty-one patients, only 4 were confirmed as positive. Of the 4 patients confirmed as positive from the analysis of saliva samples, three of them had severe symptoms of the disease, therefore, this study indicates that the use of saliva samples for detecting SARS-CoV-2 may only be useful in patients with severe symptoms. Despite this, the analysis of saliva samples represents an advantage, since patients with severe symptoms require intubation, making conventional sampling difficult for determining that the severe pneumonia they suffer is due to SAR-CoV-2. Therefore, the sampling of saliva in this type of patient makes confirmation or ruling out of infection caused by SARS-CoV-2 easier [29]. Pasomsub et al. reported the sensitivity and specificity of detecting SARS-CoV-2 from saliva samples from 200 patients through RT-PCR. The results of the saliva samples were compared with the results obtained from standard nasopharyngeal and throat swab. The sensitivity of the detection of SARS-CoV-2 from saliva samples was 84.2% [95% confidence interval (CI) 60.4–96.6%]. The specificity of detecting SARS-CoV-2 from saliva samples was 98.9% (95% CI 96.1–99.9%). The coincidence between the results of the two types of samples compared was 97.5% observed agreement (kappa coefficient 0.851, 95% CI 0.723–44 0.979; *p* < 0.001) [84]. Azzi et al. conduct the detection of SARS-CoV-2 in saliva samples of 25 patients through RT-PCR. 100% of the patients tested positive for SARS-CoV-2. The authors determined that saliva is reliable for detecting SARS-CoV-2, and it is possible that saliva also provides information on the evolution of the disease [42]. Williams et al. performed the detection of SARS-CoV-2 from saliva samples from patients who attended a clinic dedicated to detecting SARS-CoV-2 in Melbourn, Australia. Nasopharyngeal swab samples were taken from the 622 patients included in the study, these samples were analyzed and only 39 samples were positive for SARS-CoV-2. The results were subsequently confirmed in a specialized laboratory. Of the thirty-nine positive patients, thirty-three also tested positive from saliva samples using RT-PCR [85].

McCormick-Baw et al. took samples of saliva and nasopharyngeal swabs from 156 patients. 50 of the 156 patients tested positive for the nasopharyngeal swabs. Of the 50 patients who tested positive for the nasopharyngeal samples, 48 patients tested positive for the saliva samples, so the concordance of results between the two types of samples analyzed was 96%. This study used the Cepheid Xpert Xpress SARS -CoV-2 (Sunnyvale, CA) PCR test. In addition, the coronavirus genes detected in this test were genes E and N2 [86].

The results of the aforementioned studies seem to indicate that the detection of SARS-CoV-2 in saliva samples is a reliable, less invasive, and easier to perform diagnostic method. In addition to detecting SARS-CoV-2 from saliva samples using RT-PCR, other techniques that allow the development of rapid tests are being explored. The main techniques investigated for developing rapid tests are: microfluidic RT-PCR devices, antibody testing, and loop-mediated isothermal amplification (LAMP) (Table 1) [87].

### 8.1. Microfluidic RT-PCR Devices

The development of techniques that allow rapid and economical detection of viruses such as SARS-CoV-2 is useful to stop the appearance of new outbreaks. The microfluidic RT-PCR devices have advanced a lot in their complexity, from the first ones that were only capable of conducting a single procedure to current ones that can perform a great diversity of processes. These devices consist of a microchip, in which a network of microchannels for fluids is integrated. In addition to microchannels, they consist of detectors, mixers, pumps, valves, and reaction chambers. Through the integration of all these systems with the microchips, these devices can perform various protocols automatically, making this type of device an ideal means for rapid detection [88]. This type of device could be useful to control and monitor outbreaks of coronavirus such as the one presented in the *Diamond Princess* cruise ship case. This crusher had to be quarantined in the city of Yokohama, Japan, due to a rapid outbreak of SARS-CoV-2 that ended up infecting approximately 12% of the passengers and crew. The Japanese government did not have enough tests to diagnose all passengers, in addition, the gold standard for detecting SARS-CoV-2 requires trained personnel, material, and specialized equipment, and can only be performed in certified laboratories [89].

### 8.2. Loop-Mediated Isothermal Amplification (LAMP) Tests

The molecular test considered the gold standard for detecting SARS-CoV-2 is the quantitative reverse transcription PCR (RT–qPCR). The RT–qPCR has important disadvantages for its implementation in the detection of infectious diseases that spread rapidly, as in the case of SARS- CoV-2. As mentioned before, this technique requires specialized personnel, reagents, and equipment. To obtain the test result, it can take up to 2 or 3 days. The LAMP technique is based on the technology used for performing conventional PCR, therefore the RT-LAMP has several characteristics that could help overcome the disadvantages of RT–qPCR [90]. For example, the reagents used in the RT-LAMP are cheaper, the technique does not require trained personnel, can be performed at various pH and temperatures, has high specificity and sensitivity and the results can be obtained in less than one hour [91]. Lamb et al. reported a protocol for RT-LAMP where test results can be obtained in 30 min using simulated patient samples. Simulated patient samples consisted of samples from nasopharyngeal and oropharyngeal swabs, saliva, urine, and spiking serum to which portions of the nucleic sequences of SARS-CoV-2 were added. The protocol showed positive results for SARS-CoV-2 detection with high specificity and sensitivity in approximately 30 min for all samples [92]. The combination of the LAMP technique and the aforementioned microchips allows the development of devices capable of being used by ordinary people and that can provide exact results quickly. For example, Sun et al. developed a protocol for detecting SARS-CoV-2 through the LAMP technique and a microfluidic device. However, in this study they did not use samples from patients with SARS-CoV-2, the samples they used were from 5 equine pathogens. This technique was able to specifically detect the pathogens in a period of 30 min and the result could be read through a smartphone [93]. It shows us the potential of applying this type of technology in the surveillance and identification of patients with SARS-CoV-2, especially in high-risk areas such as the dental office.

### 8.3. Antibody Testing

Previous studies have shown that intraperitoneal [94] and intranasal immunization [95] elicit an immune response with the production of SARS-CoV-2-specific IgG and IgA. In addition, IgA could be detected in saliva samples. Sabino-Silva et al. proposed a salivary diagnosis of COVID-19 using specific antibodies to the SARS-CoV-2 virus [10]. To et al. demonstrated the presence of IgM and IgG in serum samples from patients infected with SARS-CoV-2 [96]. Sullivan et al. reported the presence of IgG, IgM, and IgA in saliva samples from patients with SARS-CoV-2. However, limitations of this test include possible cross-reactivity of SARS-CoV-2 antibodies with those produced against other coronaviruses. Furthermore, this test is indicated for surveillance and not for early diagnosis [97].

Saliva sampling for the diagnosis of SARS-CoV-2 has shown a greater number of advantages compared to the gold standard. Furthermore, the disadvantages found are few and not fully confirmed. Some studies report that saliva samples have greater accuracy in results compared to nasopharyngeal samples. Some studies even report that the results of saliva samples have been included as criteria to discharge patients recovered from SARS-CoV-2 [98]. The advantages and disadvantages of using saliva samples are summarized in Table 2.

Moreira, et al. published a systematic review and meta-analysis in which they compared the accuracy of standard samples (oropharyngeal and nasopharyngeal swabs) for virus detection by means of RT-qPCR with saliva samples taken from different regions of the oral cavity (deep- throat saliva/posterior oropharyngeal saliva and oral saliva) and other bodily fluids (tears, urine, sputum, and feces). The authors found that the highest virus detection accuracy was achieved with oral saliva (92.1% [95% CI: 70.0–98.3]) [99]. Lee et al. additionally performed a meta-analysis comparing the detection accuracy of SARS-CoV-2 in nasopharyngeal and oropharyngeal samples compared to saliva samples. The authors found that saliva samples were 88% accurate, while oropharynx and nasopharynx samples were 84% and 82% accurate, respectively. Only the combined oropharynx and nasopharynx samples were able to exceed the accuracy of the saliva samples by 92% [100]. Nasiri and Dimitrova performed a meta-analysis in which they compared the detection accuracy of SARS-CoV-2 between nasopharyngeal samples and saliva. In this study, the authors found no significant difference between the two types of samples [101]. Therefore, the studies mentioned above seem to indicate that saliva can substitute oropharyngeal samples for the detection of SARS-CoV-2 in suspected patients.

## 9. Conclusions

In conclusion, the authors can mention that information is being published continually and provides more scientific evidence of the mechanisms of infection of the coronavirus in oral cells and tissues. This also explains the presence of the virus in the saliva of infected people and the risk of contagion that this means. It also provides scientific evidence of the use of saliva as a biofluid for the detection, diagnosis, monitoring, and control of the spread of the virus.

## Figures and Tables

**Table 1:** Advantages and disadvantages of techniques used for developing rapid tests.

Techniques	Advantages	Disadvantages
Microfluidic RT-PCR devices	Ideal for rapid detection	Expensive Sophisticated equipment
	Useful to control and monitor outbreaks of coronavirus	
Loop-mediated isothermal amplification (LAMP)	Economic reagent Do not require trained personnel Can be performed at various pH and temperatures High specificity and sensitivity Results in less than one hour	Low throughput
Antibody testing	Indicated for surveillance	Cross-reactivity of SARS-CoV-2 antibodies with those produced against other coronaviruses
	Simple operation	Not indicated for early diagnosis
	high-throughput	Time-consuming
		Vulnerable to contamination

**Table 2:** Advantages and disadvantages of salivary sampling for the diagnosis of SARS-CoV-2.

Advantages	Disadvantages
Sampling is a noninvasive procedure.	The variation of the saliva flow can intervene in the result.
Does not cause discomfort in patients.	Salivary flow and composition can be altered by the intake of medications.
It can be applied in children or in patients with disabilities.	Oral hygiene can affect the result.
Makes it easy to take multiple samples.	
The sample can be taken by the patient, which prevents health professionals from putting themselves at risk.	
Does not need trained personnel for sampling.	
Greater accuracy compared to nasopharyngeal swabs.	
Easy to handle, transport and store.	

## Data Availability

Not applicable.

## Abbreviations

SARS-CoV	Severe acute respiratory syndrome coronavirus
MERS-CoV	Middle-East respiratory syndrome coronavirus
SARS-CoV-2	Severe acute respiratory syndrome coronavirus 2
ACE2	Human angiotensin-converting enzyme 2
RT-qPCR	Reverse transcription polymerase chain reaction
ARDS	Acute respiratory distress syndrome
HCWs	Healthcare workers
TMPRSS	Transmembrane protease
CNS	Central Nervous System
LAMP	Loop-mediated isothermal amplification
